# Social dominance hierarchy type and rank contribute to phenotypic variation within cages of laboratory mice

**DOI:** 10.1038/s41598-019-49612-0

**Published:** 2019-09-20

**Authors:** Justin A. Varholick, Alice Pontiggia, Eimear Murphy, Vanessa Daniele, Rupert Palme, Bernhard Voelkl, Hanno Würbel, Jeremy D. Bailoo

**Affiliations:** 1 Division of Animal Welfare, Veterinary Public Health Institute, University of Bern, Bern, Switzerland; 2 Department of Biology & UF Genetics Institute, University of Florida, Gainesville, USA; 3 Department of Behavioural Biology, University of Münster, Münster, Germany; 4 Department of Biomedical Sciences, University of Veterinary Medicine, Vienna, Austria; 5 Department of Pharmacology & Neuroscience, Texas Tech University Health Sciences Center, Lubbock, TX USA; 6 School of Medicine, Garrison Institute on Aging, Texas Tech University Health Sciences Center, Lubbock, TX USA; 7 Department of Civil, Environmental, and Construction Engineering, Texas Tech University, Lubbock, TX USA

**Keywords:** Social behaviour, Behavioural methods

## Abstract

A tacit assumption in laboratory animal research is that animals housed within the same cage or pen are phenotypically more similar than animals from different cages or pens, due to their shared housing environment. This assumption drives experimental design, randomization schemes, and statistical analysis plans, while neglecting social context. Here, we examined whether a domain of social context—social dominance—accounted for more phenotypic variation in mice than cage-identity. First, we determined that cages of mice could be categorized into one of three dominance hierarchies with varying degrees of dominance behavior between cage-mates, and low levels of agonistic behavior in the home-cage. Most groups formed dynamic hierarchies with unclear ranks, contrasting with recent accounts of stable transitive hierarchies in groups of mice. Next, we measured some phenotypic traits, and found that social dominance (i.e. dominance hierarchy type and degree of dominance behavior) consistently accounted for some phenotypic variation in all outcome measures, while cage-identity accounted for phenotypic variation in some measures but virtually no variation in others. These findings highlight the importance of considering biologically relevant factors, such as social dominance, in experimental designs and statistical plans.

## Introduction

Scientists studying rodents in the laboratory often attempt to account for their social needs by housing them in groups of several animals per cage^1^. Once the animals are grouped into specific cages, individual rodents can no longer be treated as independent sampling units. Treatments are therefore often applied at the cage-level and analyzed by using cage-identity as a blocking factor (e.g. by averaging results within cages or randomly selecting a single mouse per cage) or in statistical analysis plans (e.g. by including cage-identity as a random factor or covariate in a statistical model). Indeed, experimental evidence indicates that the location of a cage in the housing room is associated with weight gain^2^, retinal atrophy^3^, tumor onset^4^, and arousal sensitivity^5^—to name a few^6–8^. Thus, ignoring cage-identity could bias the outcomes of an experiment and risk artificially inflating the number of samples or replicates within a study (i.e. pseudoreplication)^9,10^. Implicit in these methods used to accommodate for shared dependencies in samples is the tacit assumption that rodents within the same cage (i.e. cage-mates) are phenotypically more similar than rodents housed in different, or neighboring, cages. Research on the social dominance of laboratory mice contests this assumption, and indicates that cage-mates with different dominance ranks have different phenotypic traits^11^. This general finding also implies that animals from different cages but with the same dominance rank (e.g. alpha ranked mice) have more similar phenotypic traits than cage-mates with different ranks (e.g. alpha, beta, gamma). These sub-group structures within a cage, such as social dominance, therefore, question common practices of averaging results across cage-mates or randomly selecting one animal per cage for data collection and analysis. Moreover, neglecting such within-cage variation may mask treatment effects and contribute to idiosyncratic results^12^.

Social dominance relationships are common for most laboratory animals. Dominance relationships are often determined by observing predictable patterns regarding which animal chases (i.e. dominant) or retreats (i.e. subordinate) during social interactions^13^. These predictable patterns may then be organized in a hierarchical fashion where each animal has a dominance rank within the group. For example, groups of laboratory mice commonly form transitive or despotic hierarchies^14,15^. Transitive hierarchies are composed of individuals with unique dominance ranks (e.g. alpha, beta, gamma), while despotic hierarchies are comprised of one individual with a defined alpha rank and whose cage-mates have undefined subordinate ranks^16^.

Social dominance ranks, and the transitive or despotic dominance hierarchies of mice, are examples of an individual’s social context within a cage. Many studies indicate that individual variability in dominance (e.g. rank and hierarchal organization) is associated with individual variability in behavior and physiology. For example, dominant mice tend to be more explorative than their subordinate cage-mates^17–23^. Also, a recent experiment demonstrated that alpha ranked mice are more susceptible to develop depression-like behavior following repeated social defeat^24^. However, no clear differences have been reported with respect to other phenotypic traits, including; anxiety^18,24–28^, learning^18,29^, social behavior^30^, sensory behavior^18,26,28^, and immune function^17,31,32^. One possible explanation for the absence of a clear difference in phenotype beyond exploratory behavior is that many studies overlook dominance rank in conjunction with different types of dominance hierarchies and assume that all cages form transitive hierarchies with a clear rank order. For example, a recent study^33^ considering both dominance rank and hierarchy, concluded that subordinate mice in highly despotic groups had significantly lower testosterone and higher levels of corticosterone than their alpha cage-mates, whereas mice living in transitive groups had similar levels of testosterone and corticosterone. Thus, individual dominance rank and the dominance hierarchy structure of the group may have a combined effect on the existence and directionality of large phenotypic differences within and between cages of mice.

The present experiment expands on our previous work^34^ and aimed to better understand the relationship between social dominance context and phenotypic variation. Dominance ranks and hierarchies were measured using the dominance tube-test (i.e. competitive exclusion task)^14,35^ for three weekly test-days at post-natal days (PND) 80, 87, and 94 in male and female RjOrl:SWISS (hereafter SWISS) mice housed in same-sex triads in standard laboratory conditions since weaning (PND 21). Agonistic behavior was also measured in the home-cage on PND 71 and 92, and in a social-reunion test on PND 94. All mice were then tested on common paradigms in pre-clinical animal research; exploration in the open field, exploration and discrimination of a novel object, anxiety-related behavior in an elevated plus-maze, and concentration of glucocorticoid metabolites in fecal samples.

We hypothesized that triads of laboratory mice would mostly form stable transitive or despotic dominance hierarchies, and that cage-mates of different dominance rank would consistently have different phenotypic traits. We also predicted that social dominance rank and hierarchy would account for more phenotypic variation than cage-identity for all measured phenotypic traits.

## Results

### Stability of social dominance hierarchies and ranks

**Figure 1:**
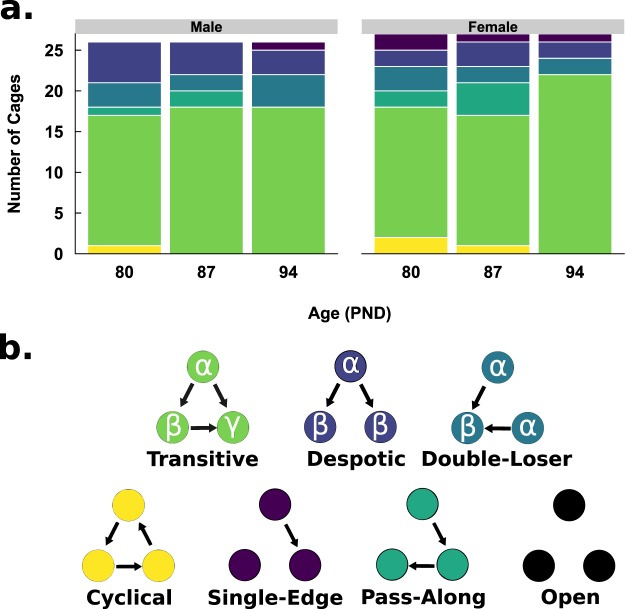
Stability of Hierarchy Type. (**a**) A stacked column chart representing the number of cages assigned to each dominance hierarchy type. Fill colors are mapped to each hierarchy type depicted in (**b**). There are no open hierarchies (black) in Fig. 1a.

The stability of dominance hierarchies and ranks, for each triad (i.e. cage-group), were measured across PND 80, 87, and 94 using the dominance tube-test. First, the triad type for each test day was measured by determining the transitivity of each group of 3 mice (i.e. a triad census of one) (Fig. 1a). For each triad, it was possible for the mice to organize into one of seven different hierarchy types (Fig. 1b). The frequency of each possible hierarchy type varied across each test day. The most frequent hierarchy type was transitive, regardless of sex. Despotic, double-loser, and pass-along hierarchy types were relatively similar in frequency, regardless of sex. No cage-groups had an open organization (no defined dominance relationships) and few had cyclical organizations (defined dominance relationships, but no ranks). Next, we determined the stability of individual rank by calculating the frequency of individuals that maintained their social dominance rank. Overall, the proportion of male and female mice displaying a stable rank was similar between sexes (Males: 33/78, 42%; Females: 31/81, 38%). Mice ranked alpha were the most stable, regardless of sex (Males: 17/33, 52%; Females: 15/31, 48%), while beta mice were the second most frequent to remain stable (Males: 11/33, 33%; Females: 12/31, 38%), followed by gamma mice (Males: 5/33, 15%; Females: 4/31, 13%).

We then combined the stability of dominance hierarchy type and the stability of rank to categorize cage-groups as either static or dynamic. Static hierarchies were composed of cages that maintained a stable dominance hierarchy type and stable individual rank assignment across test days. Dynamic hierarchies, in contrast, were composed of cages that had unstable dominance hierarchy types and unstable individual rank assignments across test days. Notably, two types of static hierarchies were categorized further; transitive or despotic. The category ‘static transitive’ was defined by cage-groups that had a stable transitive organization and stable dominance ranks (Males: 6/26; Females: 4/27). The category ‘static despotic’ was defined by cage-groups that had a stable alpha and where subordinate cage-mates switched rank at least twice (Males: 8/26; Females 8/27). All other cage-groups were assigned to the category ‘dynamic’ (Males: 12/26; Females 15/27). Thus, most groups maintained a dynamic dominance hierarchy across PND 80, 87, and 94.

**Figure 2:**
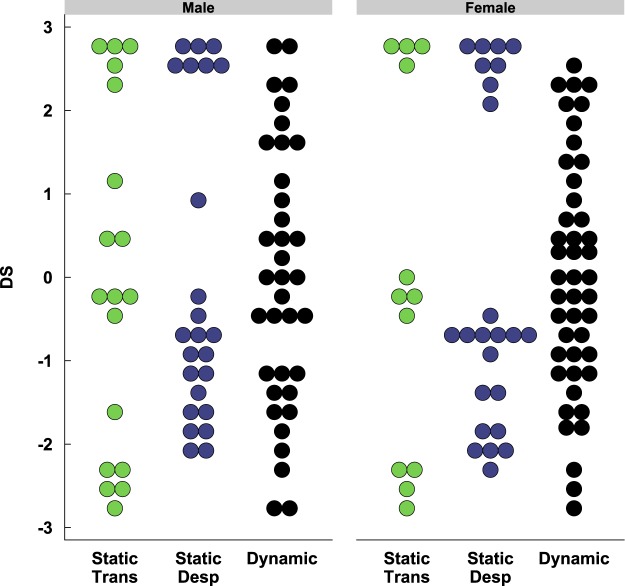
Individual DS and Dominance Hierarchy Category. DS ranges from −3 to 3, with 3 representing the highest proportion of wins and −3 representing the highest proportion of losses. Each dot represents an individual mouse.

Because dominance rankings were not comparable between the three dominance hierarchy categories—static transitive, static despotic, and dynamic—we also calculated a David’s Score (DS) as an alternative metric for characterizing dominance rank^36^. DS is a type of cardinal rank (dominance rank is an ordinal type of ranking) calculated from an individual’s proportion of wins and losses in relation to the wins and losses of its opponents; ranging from –3 to 3 for triads, where –3 represents a maximum proportion of losses and 3 represents a maximum proportion of wins^37^. All trials from PND 80, 87, and 94 were used to calculate the DS. Overall, individual DSs further confirm that the dominance hierarchy categorizations attributed to each group, from the stability of hierarchy and rank, are representative of the groups’ wins and losses in the dominance tube-test. Static transitive cage-groups show a characteristic separation of scores into three clusters (corresponding to the alpha, beta, and gamma ranks), static despotic cage-groups show two clusters (alpha ranks with subordinate cage-mates), while dynamic cage-groups show no distinctive clustering of DS but one continuous distribution over the whole range (Fig. 2).

### Frequency of agonistic behavior in the home-cage

Agonistic behavior was generally infrequent (<1% of all observed intervals) in cages of males and females during home-cage observation and in a social-reunion test and therefore no further statistical comparisons were conducted. Home-cage behavior was observed across four 1 hour long intervals across the active cycle on PND 71 and 92 (intervals: 19:00–20:00, 9:00–10:00, 13:00–14:00, and 16:30–17:30). Assessment of agonistic behavior during social reunion involved re-introduction of two cage-mates with one temporarily isolated cage-mate, immediately following the dominance tube-test on PND 94. Summary data are provided in SI Tables 1 and 2.

### Proportion of variation accounted for by social dominance

**Figure 3:**
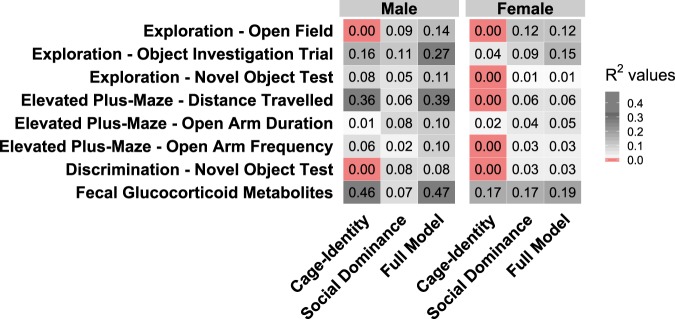
Proportions of variation accounted for by cage-assignment or social dominance. R2 values of zero/null are highlighted in red.

After measuring the dominance hierarchies and ranks in the dominance tube-test and level of agonistic behavior in the home-cage and social-reunion test, we measured behavioral and physiological responses in several tests commonly used in preclinical animal research to determine whether social dominance would account for more phenotypic variation than cage-identity. To examine this hypothesis, we calculated the proportion of variation (R^2^) explained by three different linear models; (1) *Cage-identity model*: mixed-effect model with cage-identity as a random effect; (2) *Social Dominance model*: a linear model with DS, hierarchy category, and their interaction as fixed effects; (3) *Full model*: a linear mixed-effect model with cage-identity as a random effect, DS as a fixed effect, hierarchy category as a fixed effect, and the interaction of DS and hierarchy category as a fixed effect (Fig. 3). Our initial intention was to compare two simple models of cage-identity and dominance rank; however, given the presence of different hierarchy types and instability of rank, we opted for a comparison that was more representative of our data, including the three different categories of dominance hierarchies (static transitive, static despotic, and dynamic) and dominance status as measured by DS.

We found that social dominance consistently accounted for some proportion of variation (Males: range = 0.02–0.11; Females: range = 0.01–0.17), whereas the results concerning cage-identity were inconsistent. Thus, cage-identity accounted for virtually no variation in some measures, while for other measures in males, cage-identity alone explained a large proportion of the variation (Males: range = 0.00–0.46; Females: range = 0.00–0.17).

### Relationship between social dominance and exploration behavior

**Figure 4:**
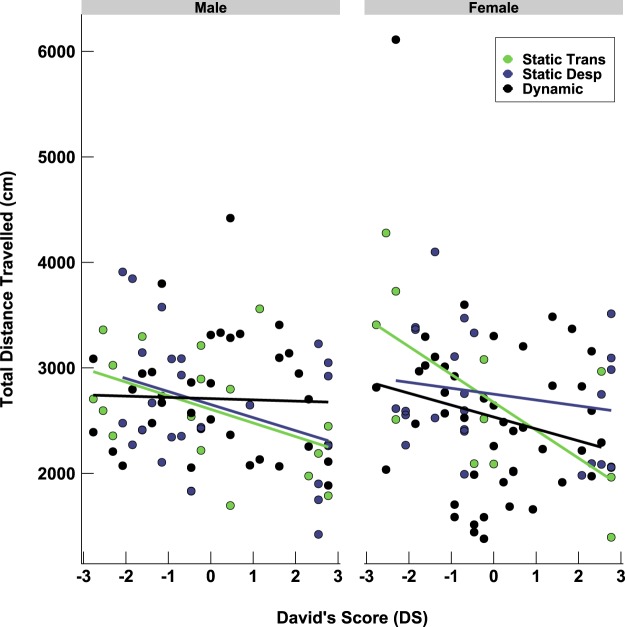
Relationship between exploration in open field and social dominance. Each point represents one mouse.

We further evaluated the relationship between social dominance and each outcome measure using the full model presented above. In all mice, exploration was measured in the open-field, an object investigation trial, and a novel object investigation test, with each test occurring on separate days from PND 98–100, and in that order. Exploration was defined as the total distance traveled for each test and all tests used the same apparatus (further described in the Methods section). Overall, there was some statistical relationship between exploration behavior and social dominance, but it was not consistent across tests (Table 1). For the measures that did show a statistical relationship between dominance rank and phenotypic outcome, subordinates were more exploratory (negative DS) than their dominant cage-mates (positive DS) (Fig. 4).

**Table 1:** Linear Mixed-Effect Models for Estimating Exploration Behavior.

Paradigm	Fixed Effect	Male			Female		
		df	F	p	df	F	p
Open Field	**DS**	**1,49**	**5.810**	0.020*	**1,75**	**8.667**	0.004*
	Hierarchy Category	2,23	0.182	0.835	2,75	0.746	0.478
	DS × Hierarchy Category	2,49	1.149	0.325	2,75	1.426	0.247
Object Investigation	**DS**	**1,49**	**7.053**	0.011*	1,51	2.548	0.117
	Hierarchy Category	2,23	1.214	0.316	2,24	0.805	0.459
	DS × Hierarchy Category	2,49	0.429	0.653	2,51	2.266	0.114
Novel Object Test	DS	1,49	1.331	0.254	1,75	0.295	0.589
	Hierarchy Category	2,23	1.369	0.274	2,75	0.015	0.985
	DS × Hierarchy Category	2,49	0.036	0.965	2,75	0.130	0.879

### Relationship between social dominance and novel object discrimination

In addition, we measured discrimination of a novel object (PND 100) following a presentation of two objects for familiarization the previous day (PND 99). The full model indicated that there were no statistical differences between mice of different DS for discrimination in the novel object test (Males: F_(1,72)_ = 1.447, p = 0.233; Females: F_(1,75)_ = 0.002, p = 0.969), between different hierarchy categories (Males: F_(2,72)_ = 0.248, p = 0.781; Females: F_(2,75)_ = 0.027, p = 0.973), nor the interaction between DS and hierarchy category (Males: F_(2,72)_ = 2.130, p = 0.126; Females: F_(2,75)_ = 1.357, p = 0.263) (SI Fig. 1).

### Relationship between social dominance and behavior in elevated plus-maze

Next, the mice were tested in the elevated plus-maze (PND 101). The outcome measures of interest were total distance traveled, time spent in the open arms, and frequency of entering the open arms. The full model indicated that there were no statistical relationships between social dominance and behavior on the elevated plus-maze (Table 2).

**Table 2:** Linear Mixed-Effect Models for Estimating Behavior in the Elevated Plus-Maze.

Paradigm	Fixed Effect	Male			Female		
		df	F	p	df	F	p
Total distance traveled (cm)	DS	1,49	0.680	0.414	1,75	1.753	0.190
	Hierarchy Category	2,23	0.977	0.392	2,75	0.617	0.542
	DS × Hierarchy Category	2,49	0.788	0.461	2,75	1.353	0.265
Frequency entering Open Arms	DS	1,49	0.154	0.696	1,51	0.663	0.419
	Hierarchy Category	2,23	0.761	0.479	2,24	1.113	0.345
	DS × Hierarchy Category	2,49	2.427	0.099	2,51	0.367	0.694
Duration in Open Arms (sec)	DS	1,49	0.009	0.923	1,75	0.743	0.392
	Hierarchy Category	2,23	0.167	0.847	2,75	0.064	0.938
	DS × Hierarchy Category	2,49	0.737	0.484	2,75	0.779	0.463

### Relationship between social dominance and fecal glucocorticoid metabolites

**Figure 5:**
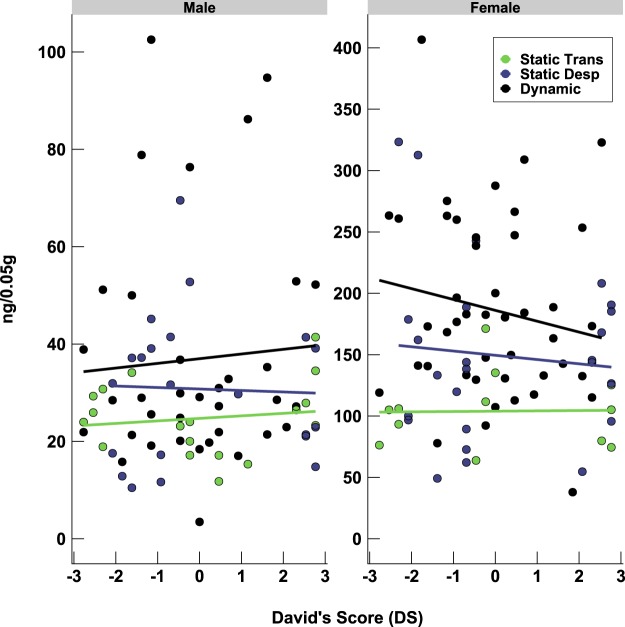
Relationship between fecal glucocorticoid metabolite levels and social dominance. The y-axis scale is different for each sex because the enzyme immunoassay for quantification of metabolites exhibits higher cross-reactivity with metabolites secreted by females than males^38^. Each point represents one mouse.

Finally, on PND 103, fecal samples were collected from individual mice to determine basal levels of glucocorticoid metabolites (sharing a 5α-3β, 11β structure)^38^, as a result of differential activation of the hypothalamic-pituitary-adrenal axis, between mice of different ranks. There was no statistical evidence that levels of fecal glucocorticoid metabolites were associated with DS (Males: F_(1,46)_ = 1.350, p = 0.251; Females_(1,51)_ = 0.724, p = 0.393) or the interaction between DS and hierarchy category (Males: F_(2,46)_ = 0.243, p = 0.786; Females_(2,51)_ = 0.313, p = 0.733). However, glucocorticoid metabolites levels differed between dominance hierarchy categories (Males: F_(2,21)_ = 1.006, p = 0.383; Females_(2,24)_ = 6.899, p = 0.004*) (Fig. 5). More specifically, hierarchies with more rank instability (i.e. dynamic) had higher levels of glucocorticoid metabolites compared to hierarchies that were more stable (i.e. static transitive).

## Discussion

Because the phenotype of a mouse partly depends on its environment, scientists often make a tacit assumption that mice within the same cage are phenotypically more similar than mice housed in different cages. We found that mice housed within a cage can have different dominance behaviors; as determined by their individual DS following interactions in the dominance tube-test. We also found that social dominance may account for differences between cages, with some cages organizing in a static transitive, static despotic, or dynamic hierarchy. After determining patterns of social dominance, we measured individual behavior on some common tests in preclinical animal research. We found that cage-identity either accounted for a large proportion or virtually no variation. In contrast, social dominance consistently accounted for some variation in all measured phenotypic traits. Overall, our results demonstrate that the validity of the assumption that individuals are more similar within cages than between cages depends on the outcome being measured.

Our results on social dominance behavior are supported by the older literature^39–41^ but contrast with more recent accounts^14^. Recent studies of social dominance behavior of mice housed in standard laboratory cages suggest that mice maintain stable (i.e. static) transitive or despotic hierarchies^14^. Although a proportion of cage-groups studied here were identified as static transitive or static despotic, a larger proportion of cage-groups were identified as dynamic. Earlier studies by Jacob Uhrich^40,41^ indicated that mice housed in laboratory cages commonly form dynamic relationships, where individuals regularly and unpredictably switch ranks. Our results using the dominance tube-test echo Uhrich’s findings. Notably, all mice in the current study were at least 80 days of age at the first dominance tube-test and had low levels of agonistic behavior. This age is well after the peak in aggression that is on average observed in mice during the rise of androgens; around 35 to 65 days of age^42^. Thus, the observed instability (or, dynamic fluctuation) cannot be explained by developmental changes in androgens, or high levels of aggression. Also, mice were housed in groups of 3, which is the smallest group size required for the measurement of transitivity and rank stability, and where groups are most likely to be stable relative to larger group sizes^16,43^. Therefore, given our sample size, age of testing, and group size, it is most likely that SWISS mice, and possibly mice in general, do not form clear and stable (i.e. static) dominance hierarchies; at least not under standard laboratory housing conditions^15,44^.

Several limitations of the dominance tube-test and the present study design should be considered for future research and generalizations of these findings^45^. First, we measured dominance in the tube-test for three weekly test-days rather than on a single day^46,47^ or across three or more consecutive days^14,35,48^. With three weekly test days we could measure the stability of dominance across time while reducing the probability of trained winners and losers associated with consecutive days of testing (i.e. task specific learning)^45^. Notably, our method identified three unique dominance hierarchy organizations. Considering our strict criteria for these unique organizations, it is unlikely that static transitive or static despotic groups were the result of learning specific to the test itself. Secondly, it is important to reiterate that the dominance tube-test measures dominance relationships, and ranks represent a stable and asymmetric dominance relationship, where one animal shows consistent offensive behavior and the partner shows consistent defensive behavior. Although experimental evidence indicates that the dominance tube-test is useful for identifying mice with stable dominance relationships, there is little evidence on whether the test can identify more dynamic relationships. Specifically, multiple studies indicate that a stable ranking in the tube-test is correlated with other measures of dominance (e.g. agonistic behavior, urine marking assay, food competition, courtship vocalization, and allogrooming)^14,27^. Unfortunately, and to our knowledge, no experiment has correlated unstable ranks (i.e. dynamic rank) in the tube-test with other measures of dominance (and agonistic behavior was too low in the current study to make this comparison). Thirdly, multiple factors can contribute to consistency in rank, for example the number of trials, time-window of measurement, degree of true dominance symmetry in the relationship, and the contextual change of a social conflict in a tube^45^. Adjusting for these factors may result in a better representation of the dominance relationship beyond identifying that an individual’s rank is unstable. However, this may also increase the chance of a learning effect specific to the dominance tube-test. Whether this is important depends on the experimental study. In sum, the dominance tube-test used in this study can identify mice with stable and static dominance relationships but has less resolution when categorizing mice with more dynamic, or unstable, dominance relationships.

Measuring the proportion of phenotypic variation accounted for by cage-assignment or social dominance (R^2^ values), we found that social dominance consistently accounted for some phenotypic variation while cage-identity accounted for virtually no variation. Specifically, cage-identity accounted for no statistical variation for 2 out of 8 of the phenotypic measures in males, and 5 out of 8 phenotypic measures in females. These findings are consistent with studies showing that social dominance may play a role in shaping phenotypic traits of individual mice^11^. Yet, they contrast with the general habit of considering cage-identity as a factor in experimental designs and statistical data analyses while social dominance is excluded. Including cage-identity as a factor in the statistical model is often advisable to reflect the nested structure of the experimental design (e.g. mice nested in cage). However, we should recognize that there are other factors, both within and between cages, which can be more contextually relevant to the mouse and should be accounted for, even if their inclusion is challenging.

Direct comparisons on social dominance hierarchy and rank with some measures of behavior and physiology revealed differences in exploration behavior and fecal glucocorticoid metabolites. First, when testing mice in the open field we found that more subordinate mice traveled further distances than more dominant mice, independent of sex. This pattern of exploration persisted after placing two objects in the open field for familiarization, but only for males. This finding adds to a mixed literature^17–21,24,27,28,49^, which assesses the relationship between exploration and social dominance. Second, our experiment indicated that fecal glucocorticoid metabolite concentrations increased as hierarchy type (static transitive, static despotic, or dynamic) became more dynamic, at least in females. This might indicate that there is a relationship between stress-related hormones and the dynamic nature, or instability, of the dominance hierarchy. A recent study^44^ housing female mice in groups of 12 indicated that they form less transitive hierarchies than males and subordinates have significantly lower levels of basal corticosterone than dominants. Further research should aim at investigating social dominance and its relation to the individual stress responses, or welfare, of female laboratory mice.

Taken together, although scientists generally acknowledge that laboratory rodents display social dominance behaviors and may form social hierarchies, such knowledge is largely neglected in experimental design and statistical analysis. Here, we demonstrate that groups of mice often form dynamic dominance hierarchies with low levels of agonistic behavior. This finding contradicts the general assumption that mice often form stable (i.e. static) and transitive dominance hierarchies but supports early studies on social dominance in laboratory mice. We also demonstrate that in contrast to cage-identity, social dominance consistently accounts for a small proportion of variation in phenotype. If generally valid, this finding highlights the importance of considering sub-group structures related to social context that may exist within cages or groups of animals when designing an experiment. Especially if the outcome of interest has a strong biological relationship with social behavior.

## Materials and Methods

### Experimental design

A total of 81 male and 81 female non-littermate RjOrl:SWISS mice (Janvier Labs, France), approximately 21 days of age, were housed in same-sex groups of 3 mice, in Makrolon Type 2 Long (325 × 170 × 140 mm) polypropylene cages. An *a priori* sample size calculation with an estimated effect size of 0.70^18^ yielded 9 groups per sex, and we tripled this number to 27 groups per sex because previous studies highlighted that around 40% of cages may have an unstable dominance hierarchy^27,34,41^. Mice were housed together since weaning and in adulthood, i.e. 10 to 11 weeks of age, the following outcome measures were collected: (a) dominance rank based on competitive ability in the dominance tube-test; (b) agonistic behavior during social reunion; (c) agonistic behavior in the home-cage; (d) behavior in an open field; (e) behavior in a novel object test; f) behavior in an elevated plus-maze; and (g) fecal samples to determine concentration of basal glucocorticoid metabolites (SI Fig. 2).

### General husbandry procedures

All subjects were kept on a 12:12 light/dark cycle with lights on at 20:00—a red light emitting diode (LED) remained on throughout the entire cycle. Temperature was 21.5 ± 1 °C and mean humidity was 53.8%. Males and females were kept in separate housing rooms. All cages contained wood chip bedding 2 cm deep (Lignocel® select), and animals had *ad libitum* access to standard rodent chow (Kliba Nafag #3430, Switzerland) and tap water. Cages, bedding, and water bottles were changed on the first day of each week in the dark phase under red light, and the body mass for each mouse was measured and recorded at this time. Cages were provided with 10 to 11 grams of nesting material (Sizzle-Pet®), and half of the unsoiled nesting material was transferred at cage change from the used cage to the new cage as this has been shown to reduce the incidence of aggression^50^. Mice were also provided a cardboard tube (Plexx EU #14152) in the home-cage to provide them with the experience of tube-like structures before encountering the tube in the dominance tube-task. All animals were ear tattooed one day after arrival for identification purposes. All animals were then fur marked with a non-toxic animal paint marker (Stoelting Co, Illinois, USA) for individual identification during video recording. One cage of males was euthanized during the first week of testing due to high levels of wounding of a cage-mate, and thus was not included in data collection.

### Dominance tube-test

We used an adapted version of the dominance tube test (i.e. competitive-exclusion task) in a round-robin tournament between cage-mates to determine individual dominance ranks. The dominance tube apparatus was a 35 cm long clear tube, 30 mm in diameter, with a small box on either end and a guillotine door in the middle of the tube (SI Fig. 4). Following a day of individual free-exploration of the apparatus for 5 minutes (PND 77), individual mice were then trained to walk through the tube with gentle nudges as necessary and opening of the door when their snout came into contact with the guillotine door (PNDs 78, 79, 86 and 93). Training involved 6 back-and-forth walks, counterbalanced for the left or right starting side. On the test day (PND 80, 87 and 94) pairs of cage-mates were simultaneously placed on opposite sides of the tube, they walked to the door, which was opened, and the cage-mates interacted. The first mouse to retreat and place its two rear paws outside the tube was recorded as the “loser” of the trial, the other mouse the “winner”. If two mice withdrew simultaneously, a “tie” was recorded (in total 4 ties out of 3,180 trials were observed). Each possible pair of cage-mates was tested in a dyadic round of 4 immediately consecutive trials, alternating the starting side of the tube. The order of testing was counterbalanced for sex, and randomized within cage using the statistical program R. Notably, some previously published methods^14,48,51^ have conducted tests on at least 3 consecutive test days, which may increase the chance of a learning effect to occur due to winner loser effects^34,45,52,53^. See SI Text 1 for further information.

### Home-cage behavior observation

Behavioral repertories of dominance were observed in the home-cage for two 24-hour periods (PND 71 and 92). The dominance behavior of each individual mouse was coded (JAV, AP, VD) in Solomon Coder (version 17.03.22). Videos were first screened for general activity over 24 hours (VD). Based on these data, four intervals were chosen in the active cycle. All cages and intervals were coded in 30 second intervals by one/zero sampling following an ethogram to measure individual dominance behavior in the home-cage (SI Table 3). Inter-rater reliability was good (ICC Median = 0.998, range = 0.831–1.0, 10% sub-sample), as was intra-rater reliability (ICC Median = 0.998, range = 0.959–1.0, 20% sub-sample)^54^. Some cages were not coded due to infrared lights poorly reflecting individual markings (28 out of 106 videos).

### Social reunion observation

Behavioral repertories of dominance were observed in the home-cage following a dyadic round in the dominance tube-test. Immediately after a pair of cage-mates completed 4 consecutive trials in the dominance tube-test (which took 4–5 minutes to complete), they were returned to the home-cage. Behavior was video-recorded and coded (JAV, AP) in BORIS (version 4.1) using an ethogram (SI Table 4). Inter-rater reliability was good (Kappa Median = 0.962, range = 0.856–1.0, 10% sub-sample), as was intra-rater reliability (Kappa Median = 0.984, range = 0.904–1.0, 20% sub-sample)^55^.

### Open field and novel object test

For all behavioral phenotyping paradigms, cage order and individual mouse within a cage were randomized, cage order was counterbalanced by sex, and only a single experimenter executed the test (JAV). Behavior in the apparatuses was scored live by Noldus Ethovision XT (version 11.5) and verified by JAV. Based on these inspections, specific trials were edited within Ethovision such that each point was accurately scored and issues associated with automated tracking were eliminated^56^. The detection settings for tracking were selected so that both the percentage of samples in which the subject was not found, and the percentage of samples skipped were less than 1% per trial. Ethovision tracked the center point of the mouse in the open-field and elevated plus-maze (EPM) and tracked the center point and nose of the mouse in the novel object test.

The open-field and novel object tests occurred over the course of PND 98–100. The open-field test is popular throughout the behavioral sciences and measures overall activity levels or anxiety-related behavior^57^. The open-field used in this study was a featureless square polycarbonate box with dimensions 45 × 45 × 45 cm and infrared backlighting. Three open-fields were placed side by side, one for each cage-mate, and diffused lighting measured at 15 lux in each field. This dim lighting is a major deviation from typical open-fields but was used because the open field test also served as the habituation trial for the novel object test. Because dim lighting was used, only information on the activity of the mouse is reported rather than anxiety-related behavior. Total distance traveled for 5 minutes was the outcome measure of interest.

The novel object test used the same apparatus as the open-field test with the addition of two objects; a bright-green polycarbonate box with large circular bumps (6.75 × 6.75 × 5.5 cm), and a dull-white polycarbonate cylinder with ridges on opposing sides (6.75 × 6.75 × 5.5 cm) (SI Fig. 3). These objects were positioned 7.75 cm away from two perpendicular walls. One day following the open field test, the mice explored the field with two identical objects for 5 minutes during a familiarization trial. Mice were then returned to their home-cage and given the same test again 6 hours later with one familiar and one unfamiliar object. The outcome variable of interest was discrimination index (DI) of the novel object (1).

1 $$DI=\frac{{\rm{duration}}\,{\rm{of}}\,{\rm{proximity}}\,{\rm{to}}\,{\rm{novel}}\,{\rm{object}}}{(\mathrm{duration}\,{\rm{of}}\,{\rm{proximity}}\,{\rm{to}}\,{\rm{novel}}\,{\rm{object}})+({\rm{duration}}\,{\rm{of}}\,{\rm{proximity}}\,{\rm{to}}\,{\rm{familiar}}\,\mathrm{object})}$$

### Elevated plus-maze

The elevated plus-maze (EPM) used in this study is similar to those used throughout mouse phenotyping^56^. This test measures exploration of open (unprotected) arms compared to closed arms (protected by high walls). Mice that spend more time in the open arms than the closed arms are assumed to be less anxious in this test situation. A total of 3 EPMs were used, each with infrared backlighting and made of polycarbonate. Each maze consisted of four arms, 30 cm in length and 6 cm wide with a center square measuring 6 × 6 cm. Two arms, opposite to each other were open, with a small lip around the perimeter 0.5 cm high, while the remaining two arms were closed, with walls 15 cm high. Overhead lighting was maintained at 600 lux. The outcome variables of interest were total distance traveled, time spent in the open arms, and frequency entering open arms.

### Glucocorticoid metabolites

Fecal boli samples were collected on experiment day 104, three days after the last phenotyping test, in the dark phase under red light from 9:00–13:00. Mice were individually housed in isolation with fresh bedding, food, and water. Individual fecal boli were collected from each cage, stored at –20 °C, and later processed (JAV and RP) according to the well-established method described by Touma and colleagues^58^. The outcome variable of interest was the concentration (ng/0.05 g feces) of glucocorticoid metabolites (sharing a 5α-3β, 11β-diol structure). For further information please see SI text 2.

### Statistical analyses

Statistical analyses were performed with R version 3.3.2. Assumptions of normality of error distribution, and homogeneity of variance were examined graphically. Based on these inspections, no transformations of data were performed. Because it is well recognized that males generally show higher levels of agonistic behavior than females, males and females were analyzed separately. Using the R package lme4^57^ three models were assessed, (i) Cage-identity: a linear mixed-effect model with only cage-identity as a random effect, (ii) Social dominance: a linear model with DS, dominance hierarchy type, and their interaction as fixed effects, (iii) Full model: a linear mixed-effect model with cage-identity as a random effect, DS as a fixed effect, hierarchy category as a fixed effect, and the interaction of DS and hierarchy category as a fixed effect. Using the MuMIn package^59^, the R^2^ value was calculated for each model to determine the proportion of variance that was accounted for by each model^60^.

### Ethical statement

This study was carried out in accordance with guidelines of the Swiss Animal Welfare Ordinance (TschV 455.1). It was approved by the Cantonal Veterinary Office in Bern, Switzerland (permit number BE56/16).

## Supplementary information

Supplementary Info

## Data Availability

All data is available via email with the corresponding author, including statistical code.
