# Supplementary material for: Social dominance hierarchy type and rank contribute to phenotypic variation within cages of laboratory mice
Source: Sci Rep. 2019 Sep 20;9:13650. doi: 10.1038/s41598-019-49612-0 (PMC6754368; doi:10.1038/s41598-019-49612-0)
Supplement: Supplementary file 1 — Supplementary Info [file 41598_2019_49612_MOESM1_ESM.pdf]

Supplementary Information Section:

Social dominance hierarchy type and rank contributes to phenotypic variation within cages of laboratory mice

Justin A. Varholick[1,2*], Alice Pontiggia[1], Eimear Murphy[1,3], Vanessa Daniele[1], Rupert Palme[4], Bernhard Voelkl[1], Hanno Würbel[1], and Jeremy D. Bailoo[1,5,6,7]

[1]Division of Animal Welfare, Veterinary Public Health Institute, University of Bern, Bern, Switzerland

[2]Department of Biology & UF Genetics Institute, University of Florida, Gainesville, USA

[3]Department of Behavioural Biology, University of Münster, Germany

[4]Department of Biomedical Sciences, University of Veterinary Medicine, Vienna, Austria

[5]Department of Pharmacology & Neuroscience, Texas Tech Health Sciences Center, Lubbock, TX, USA

[6]School of Medicine, Garrison Institute on Aging, Texas Tech Health Sciences Center, Lubbock, TX, USA

[7]Department of Civil, Environmental, and Construction Engineering, Texas Tech University, Lubbock, TX, USA

*j.a.varholick@gmail.com

Tables: 4
Figures: 4
Text: 2
Pages:10

SI Table 1 General behavior in home-cage. Mean proportion per mouse (Mean for sex divided by number of mice for each respective sex) presented for each time point.

**Behavior**	**Day 71**		**Day 92**	
	**Male** (n=57)	**Female** (n=72)	**Male** (n=57)	**Female** (n=54)
**General Activity**	62.88%	65.56%	65.56%	48.68%
**Agonistic Offensive**	3.30%	1.83%	2.07%	1.31%
**Agonistic Flee**	2.14%	1.83%	1.10%	0.81%
**Agonistic Submission**	0.63%	0.38%	0.92%	0.44%
**Allogroom**	1.95%	1.47%	1.43%	1.15%
**Stereotypy**	7.78%	16.17%	3.36%	15.36%
**Rest**	19.98%	11.60%	9.89%	7.63%
**Unseen**	16.78%	17.04%	23.38%	28.77%

SI Table 2 General behavior during social reunion. The mean frequency and duration of behaviors per mouse (Mean for sex divided by number of mice for each respective sex), across all three social reunion trials are presented. The durations of side-push were not recorded.

	**Male** (n=78)		**Female** (n=81)	
**Behavior**	**Frequency**	**Duration (sec)**	**Frequency**	**Duration (sec)**
**General Activity**	12.87	91.15	6.30	175.61
**Agonistic Offensive**	0.71	2.07	0	0
**Agonistic Chasing**	0.32	0.36	0	0
**Agonistic Mounting**	0.23	0.19	0.02	0.19
**Agonistic Side-push**	0.79	NA	1.63	NA
**Agonistic Flee**	0.35	0.46	0	0
**Agonistic Submission**	0.22	0.47	0.02	0.19
**Rest**	0.01	0.61	0	0
**Unseen**	0.85	6.83	3.38	6.31

SI Table 3 Home-cage behavior ethogram

Behaviour	Definition
General Activity	The mouse is active, but does not display a behaviour from the ethogram.
Rest	The mouse is motionless – located in the nest or somewhere else in the cage for at least 10 seconds
Agonistic Offensive (Either focal or target animal)	**Push away:** The mouse pushes aside the target animal with its snout or head, displacing the mouse. This occurs at the food hopper or on side of wall.<br>**Fighting:** Focal mouse lunges and/or bites target mouse.<br>**Mounting:** Focal mouse places two forepaws on dorsal side of target mouse from behind for at least 2 seconds.<br>**Chasing:** Focal mouse follows target mouse rapidly, while the target mouse flees.
Submission (Target animal only)	The mouse is motionless and may expose the belly to a mouse with agonistic offensive behaviour.
Fleeing (Target animal only)	The mouse shows directed movement away from a mouse with agonistic offensive behaviour
Allogrooming (Focal animal only)	The mouse's forepaws and mouth have contact with the target mouse's body trunk, shoulder region or back of head.
Receiving Allogrooming (Target animal only)	The mouse is receiving allogrooming
Stereotypy	**Bar-mouthing:** The mouse holds the cage bar in its diastema and makes a series of sham-biting or open mouth movements along the bar (for at least 5 seconds)<br>**Flipping/Looping:** Backward flip from one cage wall towards the opposite cage wall 3 successive times (with 3 or less seconds between)<br>**Route Tracing:** Repetitive tracing of a route on the cage lid or floor 3 successive times (with 3 or less seconds between)<br>**Cage-top twirling:** Spinning around the longitudinal body axis while hanging on the cage lid with the forepaws 3 successive times (with 3 or less seconds between)<br>**Jumping:** Mouse is repetitively jumping in the corner of the cage (at least 10 times within 10 seconds)
Unseen	The body of the mouse is not visible at all or cannot be distinguished during the 30 second sample point

SI Table 4 Social reunion behavior ethogram

Behaviour	Description	Modifier 1	Modifier 2
**General Activity**	The mouse is visible and active, and the behaviour being performed does not fall into the below categories or cannot be distinguished.	NA	NA
**Inactive**	The mouse is sitting or lying on the floor motionless for more than 10 seconds.	NA	NA
**Unseen**	The body of the mouse is not visible or behaviour cannot be distinguished for at least 2 seconds.	NA	NA
**Agonistic Offensive**	**Fighting:** Focal mouse lunges and/or bites target mouse. **Mounting:** Focal mouse places two forepaws on dorsal side of target mouse from behind for at least 2 seconds. **Chasing:** Focal mouse follows target mouse rapidly, while the target mouse flees.	Mouse 1<br>Mouse 2<br>Mouse 3	Fighting<br>Mounting<br>Chasing
**Agonistic Defensive**	**Subordinate Posture**: Remaining motionless and/or exposing nape or ventrum in response to agonistic offensive behaviour **Fleeing:** Running away in response to agonistic offensive behaviour	Mouse 1<br>Mouse 2<br>Mouse 3	Subordinate Posture<br>Fleeing
**Side-push** (point event, no duration)	Target mouse has at least two forepaws on side of wall or food hopper, and the focal mouse pushes aside the target mouse with its snout or whole head.	Mouse 1<br>Mouse 2<br>Mouse 3	NA
**Sniffing** (only code for females)	Focal mouse makes snout contact with target mouse head, body, or anogenital region for at least 2 seconds.	Mouse 1<br>Mouse 2<br>Mouse 3	NA

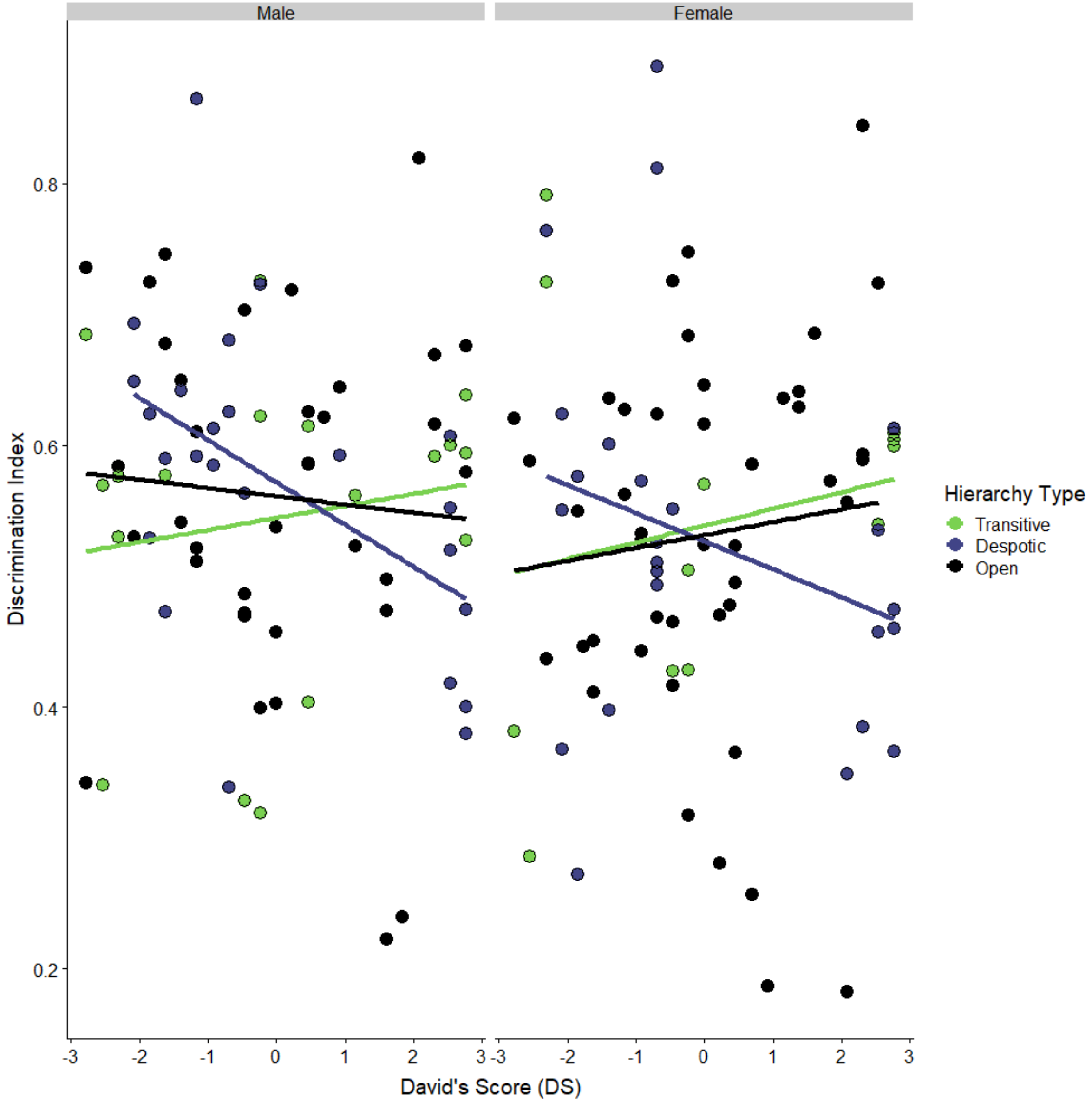

SI Fig. 1 Discrimination Index in Novel Object Test and Social Dominance

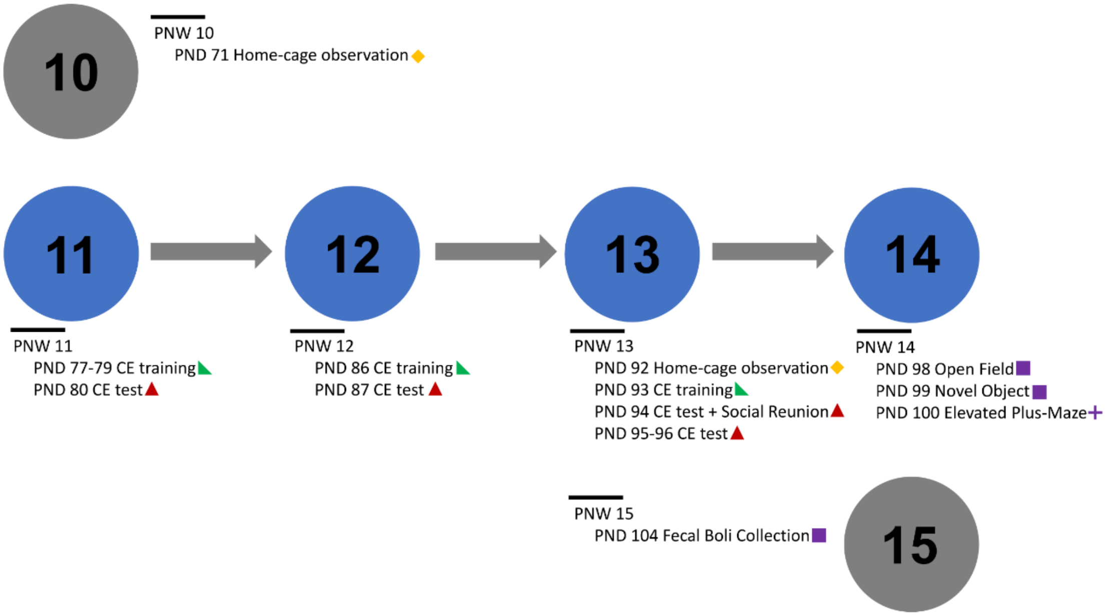

SI Fig. 2 Experimental timeline for a single batch of mice. Week 10 began at 69 days of age. PNW = Post-natal week, PND = post-natal day**.**

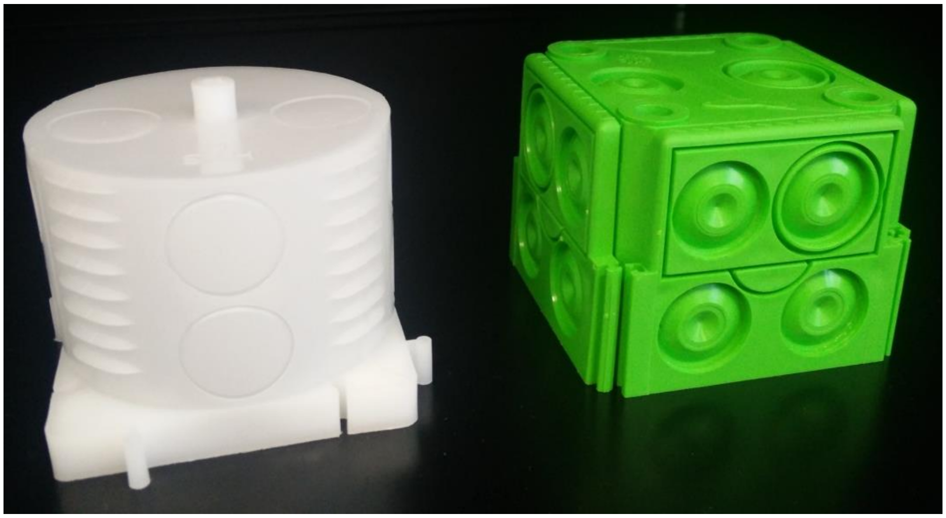

SI Fig. 3 Novel Objects

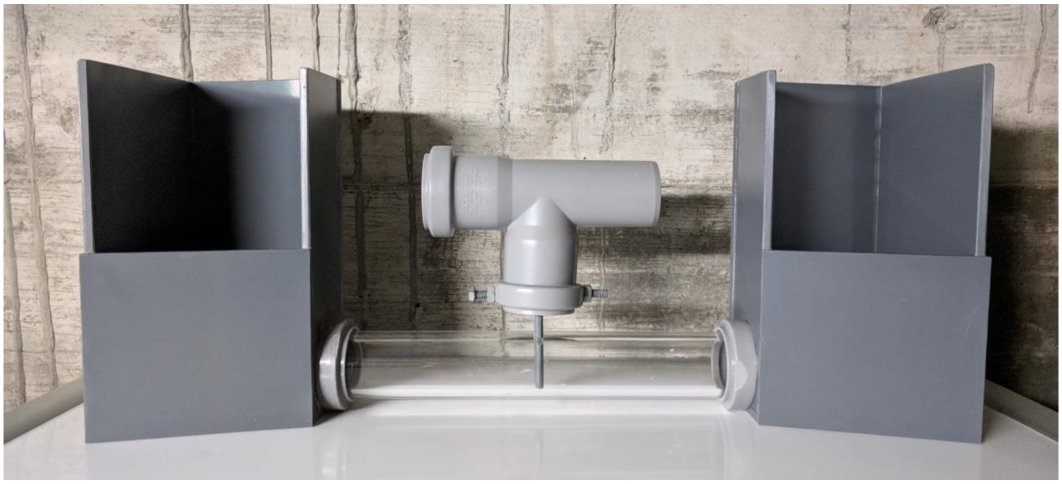

SI Fig. 4 CE Apparatus

SI Text 1 Dominance tube-test (competitive exclusion task)

General Notes

Habituation, shaping, and testing were all done in the dark phase under red light. A guillotine door is included, which slides through the middle of the tube. Notably, the door is designed for quick removal and with gaps on either side as to not completely "seal" the tube off. This allows the mice to be exposed to odor cues but limits tactile interaction. A computer randomized the order of all pairs of mice before the trials began, and the starting side of the tube was counterbalanced. The apparatus was cleaned with 70% ethanol between each dyadic round (4 trials).

Habituation (door removed)

The respective single mouse was first placed in a holding cage for 1 minute. The holding cage allows for quick and easy handling in between trials. The mouse is scooped and placed in the apparatus with nose facing the entrance of the tube. It was allowed to freely explore for 5 minutes. Each mouse traveled through the tube at least 4 times. If the mouse did not enter the tube for the first 2.5 minutes, they were gently nudged. If the mouse continually attempts to

climb out of the apparatus, they were blocked with their cage-card. After the 5 minutes is over, the mouse is returned to the holding cage and the apparatus is cleaned. The process starts over for the next mouse.

Training/Shaping

Note, the first ‘trial’ is done without a door; the door is then placed into the apparatus and used appropriately for all following ‘trials’. This is done for each training session. Individual mice were again placed in the holding cage for 1 minute. The mouse was then scooped and placed into the apparatus with its nose facing the right-side entrance of the tube. The mouse was gently nudged with a finger to enter the tube and a hand would block option to retreat from starting side. Once the mouse crossed the tube, it was nudged again to return to cross the tube and return to the starting box. The door was then placed into the middle of the tube. The mouse was gently nudged to cross the tube, and the door immediately was lifted once they came into contact with it. Once mouse fully crossed the tube, the door was closed and the mouse was nudged to cross the tube again, and the door was appropriately lifted. The mouse was then returned to the holding cage, and the apparatus reassembled without the door. This entire process was then repeated for each side such that the mouse performed 3 repetitions of this process for each side. The apparatus was cleaned between each mouse.

Test

Predetermined pairs of mice were placed in the holding cage. They were then simultaneously scooped and placed on either end of the apparatus. Once both mice got to the door, the door was lifted, and the interaction began. The first mouse to retreat within 2 minutes by placing their two rear paws outside of the tube (their starting box) was designated as the loser – this usually took less than 30 seconds. The mice were then returned to the holding cage and the

apparatus reassembled for another trial. A total of 4 trials were done for each pair of mice. After the 4th trial, the mice were returned to their home-cage and left undisturbed for 5 minutes if a partner of the next predetermined pair was in the previous test. The process was repeated for all possible pairs in the home-cage.

Social dominance scoring

First, individual wins and losses in the dominance tube-test were summed for each test day. The dominance rank for each individual on each test day was then calculated by ranking the summed frequency of wins from greatest to least, within a group of cage-mates. The triad type was then determined by organizing the assigned rank into a dominance triad (Fig. 1b). After all mice were assigned a rank for each test day, and all groups were organized into a triad type for each test day, their rank and hierarchy stability were evaluated. David's score (DS) calculated the proportion of wins and losses of each individual[42]. All calculations were done using the statistical program R v3.3.2 using the compete package v0.1 43.

SI Text 2: Glucocorticoid metabolites

Fecal boli were collected once in the dark phase under red light (c.f., Fig. 7). Starting at 9:00, mice from each cage were isolation housed in a Type 2 cages with wood shavings (Lignocel® select) 0.5 cm deep, 3-5 g of food and *ad libitum* tap water. Because the gastrointestinal transit time for corticosterone metabolites in mice has been determined to be between 4-12 hours we restricted collection of feces to a maximum of 3-3.5 hours after isolation housing, to reduce the potential confounding influence of the stress of isolation housing on measured metabolite concentration. A minimum of 10 boli per mouse was collected. Samples were immediately frozen at -20°C and later blindly processed (JV and RP) according to the

method described by Touma and colleagues[46]. A total of 159 samples were processed, 81 females.

Female mice typically have higher levels of the measured fecal glucocorticoid metabolites compared to males, which is related to sex differences in corticosterone metabolism. The enzyme immunoassay for quantification of metabolites exhibits higher cross-reactivity with metabolites secreted by females than males (Touma, C., Sachser, N., Möstl, E. & Palme, R. Effects of sex and time of day on metabolism and excretion of corticosterone in urine and feces of mice. Gen. Comp. Endocrinol. 130, 267–278 (2003)).
